# Neo-train: study protocol and feasibility results for a two-arm randomized controlled trial investigating the effect of supervised exercise during neoadjuvant chemotherapy on tumour response in patients with breast cancer

**DOI:** 10.1186/s12885-023-11284-5

**Published:** 2023-08-19

**Authors:** Eva Kjeldsted, Gunn Ammitzbøll, Lars Bo Jørgensen, Alexey Lodin, Rasmus Dahlin Bojesen, Silvia Gonzalez Ceballos, Susanne Rosthøj, Anne-Vibeke Lænkholm, Søren T. Skou, Sandy Jack, Julie Gehl, Susanne Oksbjerg Dalton

**Affiliations:** 1 Department of Clinical Oncology and Palliative Care, Zealand University Hospital, Rådmannsengen 5, Naestved, 4700 Denmark; 2 Survivorship and Inequality in Cancer, Danish Cancer Institute, Strandboulevarden 49, Copenhagen, 2100 Denmark; 3 Danish Research Centre for Equality in Cancer (COMPAS), Rådmannsengen 5, Naestved, 4700 Denmark; 4 Department of Clinical Medicine, Faculty of Health and Medical Sciences, University of Copenhagen, Blegdamsvej 3B, Copenhagen, 2200 Denmark; 5 Department of Physiotherapy and Occupational Therapy, Zealand University Hospital, Sygehusvej 10, Roskilde, 4000 Denmark; 6 Department of Physiotherapy and Occupational Therapy, The Research Unit PROgrez, Naestved- Slagelse-Ringsted Hospitals, Faelledvej 2C, 1, Slagelse, 4200 Denmark; 7 Department of Sports Science and Clinical Biomechanics, Research Unit for Musculoskeletal Function and Physiotherapy, University of Southern Denmark, Campusvej 55, Odense, 5230 Denmark; 8 Department of Surgery, Naestved-Slagelse-Ringsted Hospitals, Faelledvej 11, Slagelse, 4200 Denmark; 9 Center for Surgical Science, Department of Surgery, Zealand University Hospital, Lykkebaekvej 1, Køge, 4600 Denmark; 10 Department of Radiology, Zealand University Hospital, Sygehusvej 6, Roskilde, 4000 Denmark; 11 Statistics & Data Analysis, Danish Cancer Institute, Strandboulevarden 49, Copenhagen, 2100 Denmark; 12 Department of Pathology, Zealand University Hospital, Sygehusvej 9, Roskilde, 4000 Denmark; 13 Clinical Experimental Sciences, Faculty of Medicine, University of Southampton, University Road, Southampton, SO17 1BJ UK; 14 NIHR Southampton Biomedical Research Centre, Southampton General Hospital, MP218, Tremona Road, Southampton, SO16 6YD UK

**Keywords:** Breast neoplasms, Neoadjuvant therapy, Preoperative exercise, Prehabilitation, High intensity interval training, Treatment outcome

## Abstract

**Background:**

Prehabilitation with exercise interventions during neoadjuvant chemotherapy (NACT) is effective in reducing physical and psychosocial chemotherapy-related adverse events in patients with cancer. In preclinical studies, data also support a growth inhibitory effect of aerobic exercise on the tumour microenvironment with possible improved chemotherapy delivery but evidence in human patients is limited. The aim of the study here described is to investigate if supervised exercise with high-intensity aerobic and resistance training during NACT can improve tumour reduction in patients with breast cancer.

**Methods:**

This parallel two-armed randomized controlled trial is planned to include 120 women aged ≥ 18 years with newly diagnosed breast cancer starting standard NACT at a university hospital in Denmark (a total of 90 participants needed according to the power calculation and allowing 25% (*n* = 30) dropout). The participants will be randomized to usual care or supervised exercise consisting of high-intensity interval training on a stationary exercise bike and machine-based progressive resistance training offered three times a week for 24 weeks during NACT, and screening-based advice to seek counselling in case of moderate-severe psychological distress (Neo-Train program). The primary outcome is tumour size change (maximum diameter of the largest lesion in millimetre) measured by magnetic resonance imaging prior to surgery. Secondary outcomes include clinical/pathological, physical and patient-reported measures such as relative dose intensity of NACT, hospital admissions, body composition, physical fitness, muscle strength, health-related quality of life, general anxiety, depression, and biological measures such as intratumoural vascularity, tumour infiltrating lymphocytes, circulating tumour DNA and blood chemistry. Outcomes will be measured at baseline (one week before to 1–2 weeks after starting NACT), during NACT (approximately week 7, 13 and 19), pre-surgery (approximately week 21–29), at surgery (approximately week 21–30) and 3 months post-surgery (approximately 33–42 weeks from baseline).

**Discussion:**

This study will provide novel and important data on the potential benefits of supervised aerobic and resistance exercise concomitant to NACT on tumour response and the tumour microenvironment in patients with breast cancer, with potential importance for survival and risk of recurrence. If effective, our study may help increase focus of exercise as an active part of the neoadjuvant treatment strategy.

**Trial registration:**

The trial was registered at ClinicalTrials.gov (NCT04623554) on November 10, 2020.

**Supplementary Information:**

The online version contains supplementary material available at 10.1186/s12885-023-11284-5.

## Background

Breast cancer represents the most diagnosed cancer worldwide with estimated 2.26 million new breast cancer cases in women in 2020 [1]. Neoadjuvant chemotherapy (NACT) has shown to be effective in downsizing/downstaging large primary tumours and locally advanced breast cancer before surgery, and thereby improving the possibilities for less invasive surgery with breast-conserving and axillary-sparing procedures [2]. However, NACT is associated with several physical and psychosocial adverse events such as cancer-related fatigue, skeletal muscle deconditioning, reduced exercise capacity and impaired health-related quality of life (HRQL), which may lead to chemotherapy dose reductions and delays compromising treatment efficacy and increasing mortality [3–6].

Numerous observational studies and randomized controlled trials (RCT) have shown beneficial effects of exercise before, during and after cancer treatment on patient-reported outcomes and treatment-related adverse events [7–9], recurrence and overall survival [8, 10] across different cancers. Current evidence on exercise to improve chemotherapy completion is limited due to inconsistent findings based on secondary analyses [11, 12]. For patients with cancer receiving NACT prior to surgery, including patients with localized breast cancer, the pre-operative period is a window of opportunity to optimize the general condition of patients both physically, mentally and nutritionally [13, 14]. Pre-operative optimization has been coined prehabilitation and may improve post-operative recovery [15]. Previous RCTs testing prehabilitation interventions during NACT, with supervised in-hospital or home-based exercise, have demonstrated safety and feasibility, and improvements in important measures such as physical fitness, muscle strength, HRQL and depression [14, 16–18]. High-intensity interval training (HIIT), defined as short periods of high-intensity followed by brief recovery periods, has shown beneficial improvements in exercise capacity in several prehabilitation exercise interventions in patients with cancer [16]. Combined aerobic and resistance exercise programs have shown superior effects on cancer-related fatigue as compared to regular physical activity or yoga [19]. Adding a needs-based psychological component may improve mental wellbeing and HRQL [13], which may be important for attendance to exercise.

Evidence from preclinical studies with animal models also points to a growth inhibitory effect of aerobic exercise on the tumour microenvironment [20, 21] with e.g., increased intratumoural immune cell infiltration (in particular natural killer cells) resulting from epinhephrine spikes and interleukin-6 release [22]. In addition, aerobic exercise behaviours (treadmill/wheel running) in tumour-bearing mice and rats appear to increase microvessel density, maturity and perfusion causing reduced tumour hypoxia, increased blood-flow, and, ultimately, improved chemotherapy delivery [23]. A recent systematic review on the impact of physical activity or exercise on cancer treatment efficacy showed statistically significant improvements of treadmill/wheel running concomitant to chemotherapy on tumour volume reduction in rodents (using tumour models for breast cancer, pancreatic cancer, melanoma, Ewing sarcoma) [24]. The same review also identified seven clinical studies in humans on the topic, but all had methodological limitations as they were based on post hoc exploratory analyses or conducted as pre-post single-arm designs, which may confound the interpretations [24]. Later, a small non-randomized prospective trial (*n* = 40) including patients with oesophageal cancer showed statistically significantly higher rates of tumour regression at surgery after moderate aerobic and resistance exercise during NACT compared to usual care [25].

In patients with breast cancer, NACT enables a clinical situation to study the possible effect of exercise concomitant to chemotherapy on tumour response. However, no RCT has to date investigated if combined HIIT and progressive resistance training (PRT) during NACT can improve tumour reduction measured by magnetic resonance imaging (MRI) in patients with breast cancer. This is a highly clinically relevant outcome since tumour response is associated with improved recurrence-free survival [26] and MRI is regarded the most accurate breast imaging modality available to evaluate early treatment response to NACT [27, 28]. Furthermore, possible effects of exercise on other clinical/pathological, physical, patient-reported and biological outcomes can be examined. The overall aim of the Neo-Train RCT is to investigate the effects of supervised HIIT and PRT offered three times a week for 24 weeks during NACT, and screening-based advice to seek counselling in case of moderate-severe psychological distress, in patients with breast cancer. The primary outcome is tumour size change measured by MRI. Secondary outcomes include among others relative dose intensity of NACT, hospital admissions, body composition, physical fitness, muscle strength, HRQL, general anxiety, depression, and intratumoural vascularity, tumour infiltrating lymphocytes, circulating tumour DNA and blood chemistry.

## Methods

The study was approved by The Committee on Health Research Ethics of Region Zealand (number SJ-827), listed on the register for the processing of personal data in research in Region Zealand (number REG-109-2019, REG-074-2020), and registered at clinicaltrials.gov (NCT04623554) prior to recruitment. This study protocol describes the design of the Neo-Train RCT following recommendations from the Standard Protocol Items: Recommendations for Interventional Trials (SPIRIT) Checklist [29, 30] (Supplementary Table 1) and presents the feasibility data from a non-randomized intervention-only pilot study, which we conducted to test and adjust the study procedures for this RCT.

### Study design and setting

**Fig. 1:**
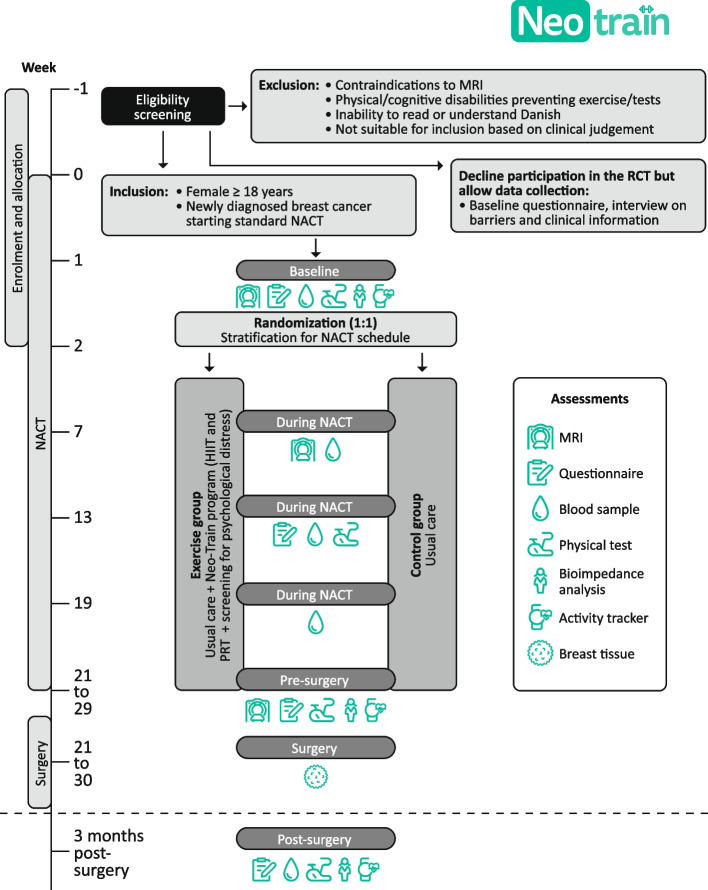
Study design of the Neo-Train randomized controlled trial (RCT). Abbreviations: HIIT: high-intensity interval training; MRI: magnetic resonance imaging; NACT: neoadjuvant chemotherapy; PRT: progressive resistance training

The study design is displayed in Fig. 1. This parallel two-armed RCT is planned to include 120 patients with breast cancer starting standard NACT. Participants will be allocated randomly (1:1) between the exercise group (Neo-Train program with supervised HIIT and PRT, and screening-based advice to seek counselling in case of moderate-severe psychological distress) and a usual care control group. Patients invited to the RCT, but who decline to participate with randomization, will be invited to complete the baseline questionnaire and contribute with clinical data collected from medical records. The purpose is to examine differences between the participants in the RCT and the patients who allow data collection regarding sociodemographics, lifestyle and the clinical outcomes. All participants will be recruited from the outpatient clinic at the Department of Clinical Oncology and Palliative Care, Zealand University Hospital, Næstved, Denmark. This outpatient clinic covers the oncological treatment of all patients diagnosed with breast cancer who are living in Region Zealand, one of five health care regions in Denmark, which has around 850,000 inhabitants and covers an area of 7,226 square kilometres.

### Inclusion and exclusion criteria

Female patients aged ≥ 18 years old with newly diagnosed histologically verified breast cancer, and scheduled to start standard NACT, will be eligible for inclusion. Patients will be excluded if they have contraindications to MRI, severe physical or cognitive disabilities preventing exercise or testing, inability to read and understand Danish, or if the oncologist, based on his or her clinical judgement, assesses the patient not suitable for inclusion.

### Recruitment procedure and randomization

Eligible patients will be identified at the multidisciplinary team conference or at the first appointment in the outpatient clinic in collaboration with the oncologists and nurses. A project team member will perform the inclusion interview. After consideration time of at least 24 h and upon written informed consent, the baseline assessment and randomization (1:1) to the exercise group or usual care control group will be performed through concealed computer-based block randomization using the electronic platform REDCAP [31]. Randomization in blocks of four stratified by anticipated ten NACT schedules (group A-J) (Table 1) will assure balanced groups of approximately same size of planned chemotherapy agents and dose intervals.

**Table 1:** Stratification groups of the planned neoadjuvant chemotherapy schedules to participants in the Neo-Train randomized controlled trial

Group	Planned neoadjuvant chemotherapy schedules according to the national guidelines of the Danish Breast Cancer Cooperative Group 2021–2023
A	Cycle 1–4: epirubicin (90 mg/m^2^) and cyclophosphamide (600 mg/m^2^) administered with three-week intervals. Cycle 5–8: twelve weekly doses of paclitaxel (80 mg/m^2^) administered on day 1, 8 and 15
B	Cycle 1–4: epirubicin (90 mg/m^2^) and cyclophosphamide (600 mg/m^2^) administered with three-week intervals. Cycle 5–8: twelve weekly doses of paclitaxel (80 mg/m^2^) administered on day 1, 8 and 15 + pertuzumab and trastuzumab
C	Cycle 1–3: epirubicin (90 mg/m^2^) and cyclophosphamide (600 mg/m^2^) administered with three-week intervals. Cycle 4–6: nine weekly doses of paclitaxel (80 mg/m^2^) administered on day 1, 8 and 15
D	Cycle 1–4: DOSE DENSE epirubicin (90 mg/m^2^) and cyclophosphamide (600 mg/m^2^) administered with two-week intervals + carboplatin. Cycle 5–8: twelve weekly doses of paclitaxel (80 mg/m^2^) administered on day 1, 8 and 15
E	Cycle 1–4: DOSE DENSE epirubicin (90 mg/m^2^) and cyclophosphamide (600 mg/m^2^) administered with two-week intervals. Cycle 5–8: twelve weekly doses of paclitaxel (80 mg/m^2^) administered on day 1, 8 and 15
F	Cycle 1–4: twelve weekly doses of paclitaxel (80 mg/m^2^) administered on day 1, 8 and 15 + phesgo. Cycle 5–8: DOSE DENSE epirubicin (90 mg/m^2^) and cyclophosphamide (600 mg/m^2^) administered with two-week intervals + trastuzumab.
G	Cycle 1–4: twelve weekly doses of paclitaxel (80 mg/m^2^) administered on day 1, 8 and 15. Cycle 5–8: epirubicin (90 mg/m^2^) and cyclophosphamide (600 mg/m^2^) administered with three-week intervals.
H	Cycle 1–4: twelve weekly doses of paclitaxel (80 mg/m^2^) administered on day 1, 8 and 15 + phesgo. Cycle 5–8: epirubicin (90 mg/m^2^) and cyclophosphamide (600 mg/m^2^) administered with three-week intervals + trastuzumab.
I	Cycle 1–4: twelve weekly doses of paclitaxel (80 mg/m^2^) administered on day 1, 8 and 15. Cycle 5–8: DOSE DENSE epirubicin (90 mg/m^2^) and cyclophosphamide (600 mg/m^2^) administered with two-week intervals.
J	Cycle 1–4: twelve weekly doses of paclitaxel (80 mg/m^2^) administered on day 1, 8 and 15 + carboplatin and pembrolizumab on day 1. Cycle 5–8: epirubicin (90 mg/m^2^) and cyclophosphamide (600 mg/m^2^) administered with three-week intervals + pembrolizumab

**Table 2:** Descriptive characteristics on sociodemographics at baseline, attendance and adherence to the exercise program among six female patients with breast cancer receiving neoadjuvant chemotherapy and included in the Neo-Train non-randomized intervention-only pilot study between 2021–2022

	n	Median	Range
**Age** (years)	6	56,5	32–71
**Body Mass Index** (kg/m2)	6	28	22–34
**Educational level**	6	-	-
High school/vocational education	3	-	-
Short or medium higher education	3	-	-
**Cohabitation status**	6	-	-
Living alone	3	-	-
Living with partner	3	-	-
Exercise duration (weeks)	3	23	13–26
Exercise attendance (sessions)	3	53	18–63
Attendance rate to exercise three times weekly (%)	3	76.8	31.0-79.7
Adherence to aerobic exercise duration (%)	3	98.4	98.1–100
Adherence to aerobic exercise intensity (%)	3	82,1	77.8–92.9
Adherence to progressive resistance training (%)	3	96.3	94.4–98.6
Total adherence rate (sum of three adherence measures) (%)	3	92.2	90.7–96.6

Three participants dropped out after ≤ 2 exercise sessions leaving only three participants for describing attendance and adherence

**Table 3:** Challenges from the non-randomized intervention-only pilot study and adjustments to the Neo-Train randomized controlled trial

Pilot design	Challenges	Adjustments to the RCT design
*Recruitment*:		
- The inclusion interview, baseline assessment and allocation should be performed before starting NACT	- Not possible in the patients who were overwhelmed by the cancer diagnosis or who had few days from the first outpatient visit to the start of NACT due to the interference of examinations such as MRI, coil insertion or long transportation	- The inclusion interview, baseline assessment and allocation will be performed from one week before to 1–2 weeks after starting NACT
- The written participant information	- Some participants believed the written participant information was too long and difficult to read	- The Patient Panel for Cancer Research at Department of Clinical Oncology and Palliative Care, Zealand University Hospital provided feedback resulting in edits and use of a more straight forward language
*Retention*:		
- No pre-defined actions to address barriers for attendance during the exercise program	- The attendance to exercise sessions varied per week during NACT due to toxicity symptoms or personal reasons	- Four scheduled telephone calls by the project manager to participants in the exercise group in week 4, 7, 13 and 19 to address barriers for adhering to the prescribed exercise - Additional telephone calls in case of no-show or repeated non-attendance - Close communication with physiotherapists on challenges
- Sample size of 100 participants planned for the RCT: 90 participants needed according to the power calculations and allowing 10% dropout (*n* = 10)	- Dropout rate of 50% (*n* = 3 out of 6 participants)	- Sample size of 120 participants: 90 participants needed according to the power calculations and allowing 25% dropout (*n* = 30).
*Exercise program*:		
- Two exercise locations and no compensation for transportation costs	- Long transportation time was the most common barrier for inclusion	- Five exercise locations - Partial compensation for transportation costs based on the distance to the exercise location
- Participants were prescribed to exercise 3 times weekly	- The invited patients and the six participants were not willing/able to exercise 3 times a week due to same-day chemotherapy administration/ examinations and long transportation, toxicity symptoms or personal reasons	- Exercise sessions will be offered 3 times a week (Mondays, Wednesdays, Fridays) - Participants will be prescribed to attend minimum 2 sessions weekly and encouraged to attend 3 sessions weekly
- Pre-scheduled progression of resistance training: Week 1–3: 65% of estimated 1RM Week 4: 70% of estimated 1RM Week 5: 75% of estimated 1RM Week 6-end: Progression after a “+2 principle” meaning that if a participant could perform ≥ 17 repetitions of good quality, the load was increased with approximately 10%	- Some participants were not able to progress in load according to the schedule due being untrained or having toxicity symptoms, which made them feel demotivated	- A more individualized resistance training program: Week 1–3: 65% of estimated 1RM Week 4-end: 65% of estimated 1RM and progression after a “+2 principle” meaning that if a participant can perform ≥ 17 repetitions of good quality, the load is increased with approximately 10%

*Abbreviations*: *MRI *Magnetic resonance imaging, *NACT *Neoadjuvant chemotherapy, *RCT *Randomized controlled trial, *1RM *One-repetition maximum

### Blinding

Due to the nature of the intervention, it is not possible to blind the participants, care providers or project team members. However, the radiologists assessing the primary outcome (MRI as per standard care) and pathologists assessing the tumour-related secondary outcomes will not be informed of the group allocation. Further, the data manager and statistician will be blinded as the group allocation will be concealed, and the interpretation of the findings will be blinded according to the approach suggested by Järvinen et al. [32].

### Neoadjuvant chemotherapy and response evaluation

All participants will receive the standard NACT, surgical and pathological procedures in Denmark as recommended by the national guidelines from the Danish Breast Cancer Cooperative Group [33–35] with any update introduced during the study period. As illustrated in Table 1, the NACT schedules will be expected to include six or eight cycles of anthracycline and taxane-based regimens with a combination of epirubicin (90 mg/m^2^) and cyclophosphamide (600 mg/m^2^) given as three to four cycles with a two-week (dose-dense) or three-week interval, and nine to twelve weekly doses of paclitaxel (80 mg/m^2^) administered on day 1, 8 and 15. Treatment with trastuzumab and pertuzumab will be given if the tumour is HER2 positive. Additionally, the platinum agent carboplatin or the immune checkpoint inhibitor pembrolizumab may be offered depending on hormonal receptor status and comorbidity. The response to NACT will be evaluated by clinical breast examination as well as MRI according to the modified RECIST criteria, and examinations of any remaining malignant cells in the breast and axillary lymph nodes after surgery. In case of lack of response, the chemotherapy agent will be changed, or the participant will be referred to early breast surgery according to guidelines. With probable chemotherapy dose delays, the time of breast surgery is expected to be within 30 weeks from starting NACT. Depending on the surgical procedure, result and receptor status, patients may be offered post-operative radiotherapy, adjuvant chemotherapy, endocrine therapy and bisphosphonate.

### The usual care control group

The control group will receive usual care with NACT and no structured pre-operative exercise guidance. These participants will be instructed to continue their normal daily activities including exercise on their own initiative and participation in municipality programs of rehabilitation if referred through usual care channels (needs-based offers typically including low-moderate intensity aerobic and resistance exercise with other patients affected by cancer or chronic diseases).

### The exercise group

The exercise group will receive the Neo-Train program consisting of a supervised exercise program and a psychological component with screening-based advice to seek counselling in case of moderate-severe psychological distress. In the following, the Neo-Train program is described considering the recommended elements from the Consensus on Exercise Reporting Template (CERT) [36], the Template for Intervention Description and Replication (TIDieR) [37] and the 13 mechano-biological determinants for describing resistance exercise [38].

#### Supervised exercise program

**Fig. 2:**
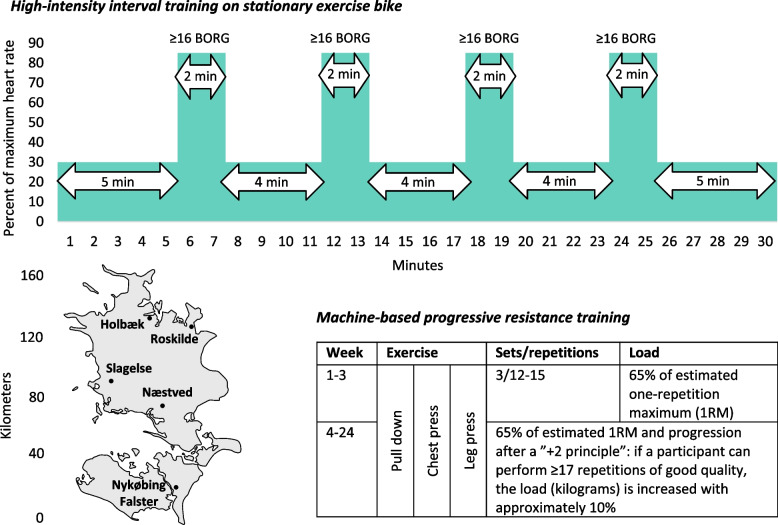
Exercise program in the Neo-Train randomized controlled trial. The sessions supervised by a physiotherapist will be offered to the exercise group three times weekly for 24 weeks from 1–2 weeks after starting neoadjuvant chemotherapy to breast surgery in groups of up to three participants

The 1-hour exercise program (Fig. 2), performed in small groups of up to three participants to stimulate motivation and social interaction, will include 30 min of HIIT on a stationary exercise bike and 30 min of machine-based PRT of large muscle groups supervised face-to-face by a physiotherapist specifically trained in the exercise manual:

- HIIT: 5 min of warm-up, four intervals of 2 min with high-intensity corresponding to ≥ 85% of maximum heart rate/≥16 on the Ratings of Perceived Exertion (BORG) scale [39] with three in-between rest periods of 4 min with low intensity, and ending by 5 min of cool-down. The heart rate will be visible to the participant and measured by a wrist-worn activity tracker.
- PRT: Three sets of 12–15 repetitions on pull down, chest press and leg press machines, respectively, with 1-2-minute rest in-between sets and around 5 min for shifting between exercises. With 2–3 s of concentric, 0 s of isometric, and 2–3 s of eccentric phases, respectively, the time under tension will be 48–90 s for each set. The physiotherapist will guide the participant to perform the exercises with a proper anatomical technique including a full range of motion for chest press and pull down, and until five degrees before full knee extension for leg press. The participant will be motivated to continue until volitional muscular failure (the participant expressing inability to continue despite encouragement). The load (kilograms) will be decided by an indirect one-repetition maximum test (1RM) performed at baseline and calculated according to the Brzycki formula:
  1RM = W*36/(37-r) where W = weight and r = repetitions.
  - Week 1–3: 65% of estimated 1RM.
  - Week 4-end: 65% of estimated 1RM and progression after a “+2 principle” meaning that if a participant can perform ≥ 17 repetitions of good quality (i.e., + 2 from 15 repetitions), the load (kilograms) is increased with approximately 10% as inspired by Courneya et al. [40] and the American College of Sports Medicine [41].

Exercise sessions will be offered three times weekly on Mondays, Wednesdays and Fridays for 24 weeks beginning 1–2 weeks after starting NACT and ending in the week before surgery (the duration may vary between 16 and up to 29 weeks depending on the NACT schedule, dose delays and timing of surgery). This will allow a recovery time in-between sessions of ≥ 48 h. Participants will be prescribed a minimum of two sessions weekly as the desired level of attendance but encouraged to attend all three weekly sessions if possible. The participants may choose one of five exercise locations across Region Zealand, which include physiotherapy departments at the university hospital, local hospitals, or a municipality rehabilitation centre. Participants will be offered partial compensation for transportation costs. The supervised program will ensure individualized exercise, starting at the outset level of the participant’s own physical ability and progressively increasing the load to accommodate exercise adaptation. This way, even untrained persons of older age, with obesity or comorbidity will be able to adhere to and benefit from the program. The program includes a standardised set of exercises using predetermined equipment. However, if toxicity symptoms or musculoskeletal restrictions prevent the participant from performing the standardised exercises at some point during the program, these may be replaced by equivalent exercises or otherwise omitted. Likewise, adjustments in intensity or duration will be allowed if a participant is not feeling well due to e.g., fatigue. If a participant is unable to attend the exercise sessions due to severe toxicity symptoms, infections, hospitalizations or personal reasons, she will not be excluded from the study but motivated to attend sessions again once possible. The physiotherapist will be instructed to register the attendance and adherence to exercise sessions, and to report any adjustments to the program in the exercise logs. On days with no sessions, participants will be recommended to be physically active 30 min daily through e.g., cycling or walking. These activities will not be systematically monitored, besides the daily number of steps, which will be monitored through the wrist-worn activity tracker. Most participants will be expected to have a Peripherally Inserted Central Catheter (PICC) in the arm for chemotherapy administration. Previous exercise trials found no PICC-related complications when performing HIIT or PRT [42, 43]. For wound healing and safety reasons, the indirect 1RM test on pull down, and PRT on pull down and chest press machines will be skipped if the PICC was inserted < 7 days ago or the participant feel pain.

#### Psychological component

Screening for psychological distress using the Distress Thermometer (DT)[44] will be performed four times: at baseline (one week before to 1–2 weeks after starting NACT), during NACT (approximately week 13), pre-surgery (approximately week 21–29) and 3 months post-surgery. The DT is a single-item tool consisting of a 0 (no distress) to 10 (extreme distress)-point Likert scale, which has been widely used to identify risk of developing psychological distress in cancer patients [45, 46]. Participants in the exercise group who score ≥ 7 (corresponding to moderate-severe distress) will be advised by a project team member to visit one of nine local Danish Cancer Society counselling centres located across Region Zealand, Denmark. Upon contact, they will be offered needs-based professional drop-in counselling free of charge based on available offers, like any other patient affected by cancer.

### Data collection

**Fig. 3:**
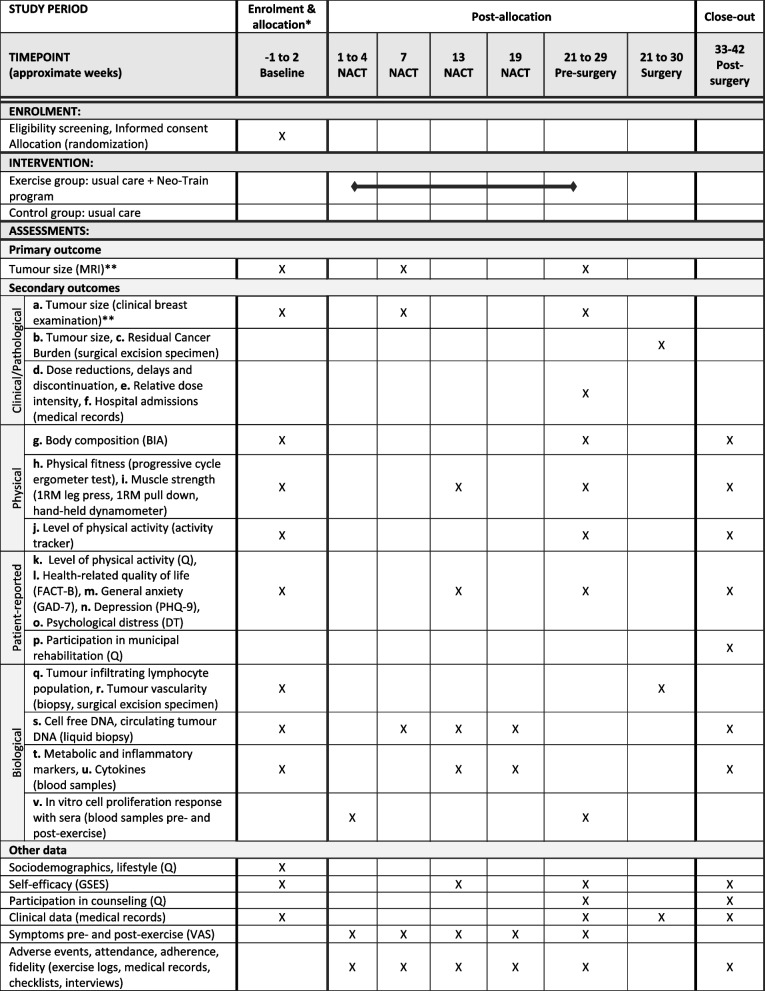
Schedule of enrolment, intervention, and assessments of the Neo-Train randomized controlled trial according to the Standard Protocol Items: Recommendations for Intervention Trials (SPIRIT) Diagram. *Enrolment and allocation will be performed between one week before to 1–2 weeks after starting neoadjuvant chemotherapy. The baseline assessment will always be completed prior to allocation (randomization). **Additional assessments of tumour size by magnetic resonance imaging and clinical breast examination may be performed depending on the individual treatment response. Abbreviations: DT: Distress Thermometer; FACT-B: Functional Assessment of Cancer Therapy - Breast Cancer scale; GAD-7: Generalised Anxiety Disorder 7-item; GSES: General Self-Efficacy Scale; NACT: neoadjuvant chemotherapy; PHQ-9: Patient Health Questionnaire-9; 1RM: one-repetition maximum

The study outcomes and assessments are illustrated in Fig. 3. Assessments will be performed at baseline (one week before to 1–2 weeks after starting NACT), during NACT (approximately week 7, 13 and 19), pre-surgery (approximately week 21–29), at surgery (approximately week 21–30) and 3 months post-surgery (approximately 33–42 weeks from baseline).

#### Primary outcome

The primary outcome is tumour size defined as change in the maximum diameter of the largest lesion in millimetre from baseline to pre-surgery measured by MRI as per standard care. The MRIs will typically be performed before start of NACT (baseline), after two chemotherapy cycles (approximately week 7) and by the end of NACT before surgery (approximately week 21–29) with additional in-between MRIs based on the individual treatment response. A project team member will extract the MRI descriptions from the medical records. Any inconsistency will be discussed with a radiologist, and if necessary, a second radiologist blinded to the group allocation will re-assess the respective images.

#### Secondary outcomes

Secondary outcomes are:

Clinical:

- *Tumour size*: measured as change in millimetre from baseline to pre-surgery by clinical breast examination performed by an oncologist or breast surgeon.
- *Tumour size*: in millimetre assessed by a pathologist after surgery from the surgically excised breast tissue.
- *Residual Cancer Burden (RCB)*: classified into RCB-0 (pathological complete response (pCR) with absence of residual disease in the breast and axilla), RCB-I (minimal residual disease), RCB-II (moderate residual disease) and RCB-III (extensive residual disease) according to the Danish Breast Cancer Cooperative Group guidelines [35] assessed by a pathologist after surgery from the surgically excised breast tissue.
- *Dose reductions, dose delays and discontinuation of NACT*: incidence in percentage from baseline to pre-surgery based on information on chemotherapy agents and dose intervals extracted from medical records.
- *Relative dose intensity (RDI) of NACT*: the delivered dose intensity ranging from 0 to 100% (actually administered mg/m^2^ in a number of weeks) as a fraction of the standard dose intensity (planned mg/m^2^ in a number of weeks based on the Danish Breast Cancer Cooperative Group guidelines [33, 34]) from baseline to pre-surgery based on information on chemotherapy agents and dose intervals extracted from medical records. RDI will be calculated similarly to the methods used by Weycker et al. [47].
- *Hospital admissions during NACT*: incidence of hospital admissions ≥ 24 h in percentage and total length in days from baseline to pre-surgery extracted from medical records.

Physical:

g. *Body composition*: changes from baseline to 3 months post-surgery in fat mass, fat-free mass and skeletal muscle mass (in kilograms) measured by bioelectrical impedance analysis (Seca medical Body Composition Analyzer 515, Seca GmbH & Co., KG, Hamburg, Germany).
h. *Physical fitness*: changes from baseline to 3 months post-surgery in indirectly estimated maximal oxygen consumption (VO^2^max) measured by a progressive cycle ergometer test [48] performed on a stationary exercise bike (Monark Ergomedic 828 E, Vansbro, Sweden). VO^2^max will be calculated using the formula VO^2^max = 0.16+(0.0117xMPO) where MPO is maximal power output in watts [48]. The test has been used previously to measure aerobic capacity in cancer patients during chemotherapy [49].
i. *Muscle strength*: changes from baseline to 3 months post-surgery in muscle strength measured by an indirect 1RM test on pull down (BH L110 Lat Pulley, BH Fitness, UK) and leg press (ARTIS® Leg Press, Technogym, Cesena, Italy), and in maximal isometric handgrip strength measured as the maximum kilograms obtained from three measurements on each hand by a hand-held dynamometer (DHD-1 Digital Hand Dynamometer, Saehan Corporation, South Korea).
j. *Level of physical activity*: changes from baseline to 3 months post-surgery in daily step count measured by a wrist-worn activity tracker (Vivosmart 4, Garmin International Inc., Olathe, KS, USA). Participants in the exercise group will wear the activity tracker during the entire study period, while the control group will wear it three times (baseline, before surgery and 3 months post-surgery) for a period of seven to fourteen days each time.

Patient-reported:

k. *Level of physical activity*: changes from baseline to 3 months post-surgery in self-reported hours per week of exercise (e.g., running, gymnastics, ball games) and everyday physical activity (e.g., cycling, walking, gardening), and per day of sedentary time (not including sleep) by three items with categorical response options on number of minutes/hours in a questionnaire.
l. *Health-related quality of life*: changes from baseline to 3 months post-surgery assessed on the Functional Assessment of Cancer Therapy - Breast Cancer scale (FACT-B) [50] in a questionnaire.
m. *General anxiety*: changes from baseline to 3 months post-surgery assessed on the Generalised Anxiety Disorder 7-item scale (GAD-7) [51] in a questionnaire.
n. *Depression*: changes from baseline to 3 months post-surgery assessed on the Patient Health Questionnaire-9 scale (PHQ-9) [52] in a questionnaire.
o. *Psychological distress*: changes from baseline to 3 months post-surgery assessed on the DT [44] in a questionnaire.
p. *Participation in municipal rehabilitation*: incidence of referral and participation in percentage from surgery to 3 months post-surgery and description of attended activities assessed by a questionnaire.

Biological:

q. *Tumour infiltrating lymphocyte (TIL) population*: percentage of TILs evaluated by a pathologist from the diagnostic needle biopsy at baseline and the surgically excised breast tissue after surgery using histopathological analyses by immunochemistry and on hematoxylyn and eosin (H&E)-stained sections of the Formalin Fixed Paraffin Embedded (FFPE) tissue blocks.
r. *Tumour vascularity*: analyses of vascularity density and structure evaluated by a pathologist from the diagnostic needle biopsy at baseline and the surgically excised breast tissue after surgery using histopathological analyses by immunochemistry and on H&E-stained sections of the FFPE tissue blocks.
s. *Cell free DNA and circulating tumour DNA*: changes from baseline to 3 months post-surgery (liquid biopsy).
t. *Metabolic and inflammatory markers*: changes from baseline to 3 months post-surgery in glucose, hba1c, zinc, magnesium, phosphate and C-reactive protein collected from blood samples, which will be analysed after usual care guidelines immediately after collection.
u. *Cytokines*: changes from baseline to 3 months post-surgery in cytokines from blood samples.
v. *In vitro cell proliferation response*: with sera collected from blood samples before and after exercise two times (during the first four weeks of NACT and within six weeks before breast surgery in a subgroup of participants).

#### Other data

Sociodemographic characteristics (marital status, cohabitation status, having children, education level, and work market affiliation), lifestyle data (smoking status, alcohol consumption), self-efficacy on the General Self-Efficacy Scale (GSES)[53] and participation in counselling at local Danish Cancer Society centres will be collected in a questionnaire. Clinical data extracted from the medical records will include comorbidity status and prescription medications, tumour characteristics, planned and received cancer treatment, routine blood work and toxicity symptoms. Immediately before and after each exercise session, the exercise group participants will report symptoms of fatigue, nausea, pain, dizziness and mood as a handwritten mark on a 10 centimetre paper-version of the Visual Analogue Scale (VAS) [54].

### Adverse events

Any suspected side effects or harm associated with the intervention or assessments (reported by participants, observed by physiotherapists or project team members in medical records) will be registered and monitored prospectively. Chemotherapy-related side effects and temporary mild discomfort from blood sampling, muscle soreness or joint stiffness after exercising will be expected, especially in untrained individuals. All other adverse events (AEs) will be listed using descriptive statistics. AEs resulting in hospitalization, prolonged hospital care, persistent disability, death or which are life-threatening will be defined and reported as serious adverse events (SAEs).

### Attendance, adherence and fidelity

In the intervention group, *the attendance rate* for each participant will be calculated as the number of attended sessions divided by the number of prescribed sessions equal to two times a week from the first exercise session (1-2 weeks after starting NACT) to one week before surgery or withdrawal. Sessions cancelled by the exercise location or the participant due to same-day chemotherapy administration/examinations will not count as non-attendance. *Adherence* to the prescribed exercise will be calculated separately for three measures, inspired by Witlox et al. [55]: 1) *the duration of aerobic exercise *(participating in HIIT and rest periods, 20 minutes =adherent), 2) *the intensity of aerobic exercise* (week 1-3 and the first week after an exercise break in case of e.g., hospitalization: BORG scale ≥14 in each 2-minute HIIT interval =adherent; week 4-end: BORG scale ≥16 and/or ≥85% of maximum heart rate in each 2-minute HIIT interval =adherent), 3) *the number of sets, repetitions and load of PRT* (week 1-end: three sets of 12-15 repetitions with a load of ≥65% estimated 1RM =adherent). The three measures will be summed in *a* *total adherence rate.* *Fidelity, *referring to how the physiotherapists deliver the intervention*, *will be described using field observations with checklists, and qualitative interviews with physiotherapists and participants regarding acceptance, motivation and barriers for exercise. Detailed examinations of possible associations between patient characteristics and high or low attendance and adherence, and fidelity measures, will be published separately in a process evaluation.

### Data storage

The data including electronic surveys sent to participants will be collected and stored using the electronic platform REDCAP [31]. If participants prefer a paper-version, a project team member will register the answers from the survey in REDCAP immediately after completion. The blood samples will be stored in a research biobank in Region Zealand Biobank. The surgically excised breast tissue will be collected under the auspices of the clinical biobank Danish Cancer Biobank/Bio and Genome Bank Denmark.

### Patient involvement

In the design phase, we established a patient panel with five patients receiving NACT for breast cancer (median age 48 years, range 36 to 77 years; median BMI 32 kg/m^2^, range 23 to 50 kg/m^2^) to inform the planning of the pilot study. The panel participants were interviewed twice, in December 2019 and February 2020, using individual semi-structured qualitative interviews on exercise type, location and potential barriers. The participants preferred combined aerobic and resistance exercise performed in groups under supervision in a safe environment such as local hospitals. These ideas were integrated in the study design.

The Patient Panel for Cancer Research at the Department of Clinical Oncology and Palliative Care, Zealand University Hospital [56] also provided feedback on the written participant information for the RCT resulting in edits and use of a more straight forward language.

### The pilot study and adjustments

To inform this RCT, we conducted an intervention-only non-randomized pilot study including six patients with breast cancer from the same outpatient clinic. The aim was to test and adjust the study procedures for this RCT. Participants were recruited between 2 March and 16 April 2021 with end of study on 4 February 2022 (3 months post-surgery for the last participant). Of the 22 patients screened for eligibility, 7 were excluded based on respectively contraindications to MRI (*n* = 1), comorbidity preventing exercise/tests (*n* = 4), not possible to conduct the baseline assessment prior to NACT (*n *= 1), and participation in a clinical drug trial/not receiving standard NACT (*n *= 1). The recruitment rate was 40% (6 out of 15 invited patients) with long transportation to an exercise location (*n *= 6) and not willing to exercise three times weekly (*n *= 3) as the main reasons for declining. The six participants had a median age of 58 years (range 32-71 years), a median BMI of 28 kg/m^2^ (range 22-34 kg/m^2^) and represented different educational levels and cohabitation status (Table 2). Three participants dropped out after ≤2 exercise sessions due to lack of energy to exercise three times weekly (*n* = 2) and the scheduled exercise time slot (*n *= 1). Among the remaining three participants (retention rate 50%), the median exercise duration was 23 weeks (range 13-26 weeks) with a median of 53 attended sessions (range 18-63 sessions) and a median *attendance rate* of 76.8% (range 31.0-79.7%) based on exercise prescribed three times weekly. The primary reasons for non-attendance were fatigue, diarrhoea and same-day chemotherapy administration. The median *total adherence rate* was 92.2% (range 90.7-96.6%), and the primary reason for non-adherence was exhaustion (Table 2). No AEs during exercise or assessments were reported. In general, both participants and physiotherapists reported positive feedback on the exercise program and assessments including acceptance of the activity tracker. Despite acceptable attendance, adherence and high satisfaction among the three remaining participants, the low recruitment and high dropout rates gave reason to change some study procedures. Table 3 illustrates the main challenges from the pilot study and the adjustments to the RCT in attempt to increase recruitment, retention, attendance and adherence to the exercise program.

### Plan to promote retention of participants

To promote retention of participants in both groups, the assessments will be planned in close collaboration with the participants and aimed to be scheduled on days of other appointments at the hospital to minimize transportation. Furthermore, all participants will be provided verbally with the physical tests results after completion and a print-screen of body composition. In the exercise group, the supervised exercise program is designed with individualised but group-based exercise offered in local areas, allowing for some flexibility and with partial compensation for transportation costs. To increase motivation and address potential barriers for attendance, a project team member will contact the participants four times by telephone or face-to-face in the outpatient clinic in week 4, 7, 13 and 19, and more often if needed.

### Power considerations

No relevant published literature was available for power calculations of tumour response to NACT with supervised exercise. Instead, power calculation was based on tumour response to NACT in usual care from the same outpatient clinic [57]. Among 105 patients available for our analysis, 35 patients (33%) had a tumour size of 0 millimetre (radiologic complete response) and 70 patients had a median tumour size of 17 millimetre at the MRI pre-surgery, respectively. Using a two-part mixture model and adjusting for log-transformed baseline tumour size, logistic regression was used to estimate the probability of radiologic complete response, and next a linear regression was used to model the log-transformed size of the tumour among those having a tumour size larger than zero. Based on the data from [57], we assumed a decrease in the odds of a tumour size of 0 millimetre of 7% per doubling of baseline tumour and an increase in tumour size of 80% per doubling among those having a tumour size larger than zero. The power was found by simulation using these assumptions for the outcome and simulating baseline tumour size from the observed baseline distribution: With 45 patients in each group we obtain a power of 80% with a significance level of 5% to detect an odds ratio of 1.5 of tumour size 0 millimetre combined with a 38% reduction in tumour size for those with a tumour size larger than zero at the MRI pre-surgery in the exercise group compared to the usual care control group. At first, we planned to include 100 participants allowing for 10% dropout (*n *= 10). Based on 50% dropout in the pilot study, we adjusted our planned sample size from 100 to 120 participants before starting the RCT, thereby allowing a dropout rate of 25% (*n *= 30). As the study proceeds and the actual drop-out rate can be assessed, the total participant number may be modified to take this into account.

### Analyses

A detailed statistical analysis plan will be uploaded to the platform Open Science Framework (osf.oi) before unblinding group allocation. Data from the patient-reported questionnaires will be scored according to manuals from the respective validated scales. Descriptive statistics will be used to present patient-reported and clinical characteristics for both groups with percentages, mean (standard deviation) for normally distributed data and median (range) for skewed data. All outcomes will be analysed using intention-to-treat analyses based on all the randomized participants.

The primary outcome (comparison of pre-surgery tumour size by MRI between groups) will be analysed adjusted for baseline tumour size using a two-part mixture model as described in the power calculation above. Several different models will be used for the analysis of the secondary outcomes (clinical/pathological, physical, patient-reported and biological measures between groups) due to the different nature of these outcomes. These models include the two-part mixture model, linear regression, Poisson regression (or eventually negative binomial and zero-inflated Poisson), ordinal and logistic regression. To account for repeated measures, mixed models will be applied for the linear models whereas Generalized Estimating Equations will be applied for the non-linear models. In these models, the baseline outcome will also be considered an outcome, however, constraining the level at baseline to be equal in the two groups due to randomization. As the mixed models implicitly accounts for missing data assuming data are missing at random (MAR), missing data techniques will only be applied for the non-linear models in case there is a substantial amount of missing data.

Two separate per protocol analyses will be performed to further elaborate the effects of the performed exercise on the primary and secondary outcomes. These will be based on attendance three or two times a week, respectively, and including participants with *an attendance rate* of ≥75% and *a total adherence rate* of ≥75%. If possible, a third analysis will be performed including participants with high adherence to HIIT (*an attendance rate* of ≥75% and a sum of *adherence to aerobic exercise duration* and *adherence to aerobic exercise intensity* of ≥75%) since this part of the exercise program may be hypothesized to have most effects on tumour size changes as compared to PRT. However, as this analysis might be biased due to post-randomization time-dependent confounding, a causal analysis based on inverse probability of treatment weighting [58] will be attempted. The weights will be estimated using the information gathered at the exercise sessions as well as baseline data and outcomes measured prior to non-adherence.

Planned explorative analyses including participants from the exercise group will include self-reported fatigue, nausea, pain, dizziness, and mood from before and after the exercise sessions. These plan to be analysed by mixed linear models.

Participants in the RCT will be compared to the patients, who decline participation in the RCT but allow data collection, using chi-square tests with a significance level of 5% of the sociodemographics, lifestyle, psychosocial and clinical characteristics from the baseline questionnaire and medical records. Furthermore, results on the clinical/pathological outcomes from medical records will be compared between groups using the same methods as described above.

### Dissemination of results

The results will be submitted for publication in international peer-reviewed scientific journals, presented at conferences and communicated via social and general media. Any positive, negative or inconclusive results will be disclosed. The reporting of findings will follow the Consolidated Standards of Reporting Trials (CONSORT) statement [59], the CERT [36] and TIDieR [37] templates. The primary outcome tumour size by MRI will be reported in the primary paper with the secondary clinical/pathological outcomes and possibly the biological outcomes of TILs, tumour vascularity and cell-free DNA/circulating tumour DNA. The remaining secondary outcomes, exploratory analyses of symptoms before-after exercise and a process evaluation will be reported in separate papers.

### Trial status

Recruitment to this RCT started on 26 June 2021. With 95 included participants on 17 July 2023, and an expected recruitment rate of 50%, recruitment is anticipated to end in January 2024 with the end of study 3 months post-surgery for the last participant in October 2024.

## Discussion

Current evidence demonstrates beneficial effects of exercise for patients with cancer but with very few randomized studies reporting on clinical outcomes and with clear descriptions of the interventions. The Neo-Train RCT will contribute to the growing field of research on prehabilitation by examining if supervised HIIT and PRT during NACT for breast cancer can improve tumour response and other highly clinically relevant secondary outcomes. The inclusion of multiple biological markers with different molecular pathological analyses of the collected blood and tissue will be important to examine if there are beneficial effects of the exercise program and, crucially, to help understand the underlying mechanisms. Other studies are underway with the aim of investigating improvements of exercise during NACT for breast cancer on tumour-related primary outcomes such as the Neo-ACT trial testing the effects of home-based HIIT and PRT supported by a mobile application compared to usual care on pCR [60], and the three-arm BENEFIT-trial, which is comparing the effects of aerobic exercise vs resistance exercise vs usual care on tumour size measured by mammography/sonography (clinicaltrials.gov NCT02999074). Our findings will, together with these up-coming studies, contribute to describe different dose, intensity and type of exercise on NACT efficacy in patients with breast cancer.

Our study has several strengths including the randomized design with blinding of the assessor of the primary outcome. The supervised machine-based sessions with exercise logs completed by physiotherapists will enable precise descriptions of the performed exercise and ensure transparent documentation of the intervention. The monitoring of heart rate and BORGs during HIIT will provide accurate estimates of the level of exertion, making it possible to give assumptions on a dose-response relationship between exercise amount and tumour size. The biological outcomes such as evaluation of TILs, which has shown to be associated with higher pCR rates and improved prognosis after NACT [61], will bring novel detailed insights on the underlying mechanisms of tumour elimination with added exercise. Further, the repeated collections of liquid biopsy will show changes in circulating tumour DNA during exercise and, in general, during the course of NACT. The screening-based advice to seek psychological counselling might help persons in most need who may be reluctant to seek help, which has the potential to alleviate the known disruptions of a breast cancer diagnosis and treatment on the mental wellbeing. Another strength will be the analyses of the patients declining to participate in the RCT but who allowed data collection from medical records. These findings will not only provide important knowledge of the barriers for participation to be used in the design of future prehabilitation interventions, but they will also allow us to compare the chemotherapy delivery and tumour response to participants in the RCT, which will help evaluate the generalisability of our findings. There is a social gradient in physical activity in both the general population and among cancer survivors with higher odds of a sedentary lifestyle among persons with short compared to long education [62]. Furthermore, the decision not to participate in exercise trials has shown to be influenced by education, social support and exercise habits in patients with cancer [63, 64]. Our study has been designed to also support inclusion of untrained persons with lower physical fitness, who are obese or have chronic diseases by offering individualized exercise in fixed conditions (e.g., group-based sessions with simple machine-based exercises under supervision). As these persons are overrepresented among patients with low socioeconomic position, we hope to ensure less selection by social and human resources and through this minimize social inequality in breast cancer outcomes.

The study has some limitations. Participants will be recruited from a single centre, and it will not be possible to blind the participants or care providers due to the nature of the intervention. Attendance and adherence to the exercise sessions will be the main challenges as it has been in previous prehabilitation interventions [65]. We have attempted to address this through a high degree of patient involvement in the design phase and by offering a structured, but flexible group-based exercise program in local areas with partial compensation for transportation costs and pre-scheduled telephone calls to increase motivation. In a meta-analysis on recruiting and retaining patients with breast cancer in exercise trials, week-to-week flexibility in intervention appointments was suggested as a possible strategy to minimize dropout [66]. We will offer exercise sessions on three weekdays, which will allow both high attendance and flexibility, which will be crucial due to competing appointments at the hospital and expected toxicity symptoms particularly in the first days after chemotherapy administration. Exercise contamination in the control group will be a possible challenge, since we offer no exercise intervention for the control group after the intervention period [67]. Although the use of wrist-worn activity trackers may have some validity issues related to the accuracy of heart rate measurements and step count [68, 69], these devices and questionnaires on physical activity, sedentary time and participation in municipal rehabilitation will be important to describe the daily physical activity in both groups.

Prehabilitation with exercise is an excellent opportunity to involve patients actively in their own treatment. Along with improvements of prehabilitation programs on patients’ physical and mental wellbeing [14, 16–18], it would have important implications for survival and risk of recurrence, if exercise can also improve tumour response to NACT. Currently, the exercise guidelines and services vary substantially across cancer care providers internationally with limited implementation of exercise as standard clinical practice in oncology [70]. If supported by our results, the Neo-Train program, using basic equipment available in most training centres, could be implemented with limited costs. Perhaps considering needs-based supervision with more support for patients of older age, with multimorbidity and obesity, who after cancer treatment often not participate in physical activity [71].

### Supplementary Information

**Additional file 1.**

## Data Availability

Not applicable.

## Abbreviations

AE: Adverse events
BORG: Ratings of Perceived Exertion scale
ER: Estrogen receptor
FFPE: Formalin Fixed Paraffin Embedded
HER2: Human epidermal growth factor receptor 2
H&E: Hematoxylyn and eosin
HIIT: High-intensity interval training
HRQL: Health-related quality of life
MPO: Maximal power output
MRI: Magnetic resonance imaging
NACT: Neoadjuvant chemotherapy
pCR: Pathological complete response
PICC: Peripherally Inserted Central Catheter
PRT: Progressive resistance training
RCT: Randomized controlled trial
RDI: Relative dose-intensity
SAE: Serious adverse event
1RM: One-repetition maximum
